# Effectiveness of Physical Activity in Primary Prevention of Anxiety: Systematic Review and Meta-Analysis of Randomized Controlled Trials

**DOI:** 10.3390/ijerph19031813

**Published:** 2022-02-05

**Authors:** Patricia Moreno-Peral, Alberto Pino-Postigo, Sonia Conejo-Cerón, Darío Bellón, Beatriz Rodríguez-Martín, Vicente Martínez-Vizcaíno, Juan Ángel Bellón

**Affiliations:** 1 Biomedical Research Institute of Malaga (IBIMA), 29010 Málaga, Spain; predictmalaga@hotmail.com (P.M.-P.); jabellon@uma.es (J.Á.B.); 2 Prevention and Health Promotion Research Network (redIAPP), 08007 Barcelona, Spain; beatriz.rmartin@uclm.es; 3 Faculty of Medicine, University of Málaga (UMA), 29010 Málaga, Spain; apinomlg@gmail.com; 4 PROFITH “PROmoting FITness and Health Through Physical Activity” Research Group, Department of Physical and Sports Education, Faculty of Sport Sciences, Sport and Health University Research Institute (iMUDS), University of Granada, 18011 Granada, Spain; dariobellon@ugr.es; 5 Faculty of Occupational Therapy, Speech Therapy and Nursing, Talavera de la Reina, Toledo, University of Castilla-La Mancha (UCLM), 13001 Ciudad Real, Spain; 6 Social and Health Care Research Center, University of Castilla-La Mancha, 16002 Cuenca, Spain; vicente.martinez@uclm.es; 7 ‘El Palo’ Health Centre, Health District of Primary Care Málaga-Guadalhorce, Servicio Andaluz de Salud (SAS), 29018 Málaga, Spain; 8 Department of Public Health and Psychiatry, University of Málaga, 29010 Málaga, Spain

**Keywords:** anxiety disorders, exercise, primary prevention, systematic review, meta-analysis

## Abstract

The aim of this study was to evaluate the effectiveness of physical activity in the primary prevention of anxiety. A systematic review of randomized controlled trials (RCTs) was performed. RCTs were searched in seven electronic databases. We included RCTs that assessed either the incidence of anxiety or the reduction of anxiety symptoms which excluded participants with baseline anxiety. Measurements were required to have been made using validated instruments. Objective or subjective (with validated questionnaires) verification of the performance of physical activity was required. Three reviewers carried out the search, selection, data extraction, and risk assessment of Cochrane Collaboration’s tool simultaneously and independently, reaching an agreement in their discrepancies by consensus. In addition, a meta-analysis of fixed-effects model was carried out. Three RCTs met inclusion criteria, comprising 350 patients from 3 different countries. A meta-analysis was performed using five comparisons extracted from the selected studies, and the pooled standardized mean difference (SMD) was −0.18 (95% CI: −0.44; 0.07), *p* = 0.158. The heterogeneity was irrelevant, I^2^ = 17.7% (*p* = 0.30). There is no evidence that anxiety can be prevented through physical activity, although the quality of evidence was very low.

## 1. Introduction

Currently, there are around 300 million people suffering from anxiety worldwide [1]. The disease burden attributable to anxiety disorders, measured in years lived with this disability (YLD), ranks eighth (women) and thirteenth (men) in the world [1]. This burden of disease had a 12.4% and 13.6% relative increase for women and men, respectively, in YLD between 2007 and 2017 [2]. Evidence shows anxiety increases mortality by 43% (relative increase), whether or not patients suffer from any other concomitant diseases [3]. Suicide relative risk in these patients rises up to 117% compared to the general population [4].

In order to reduce the burden attributable to anxiety disorders, two main approaches can be described. The first one consists on treating the illness once it affects the patients’ health. This is known to be effective [5,6], although not every patient has access to early and adequate treatments [7].

The second one, which is prevention, can eliminate completely any kind of consequence an anxiety disorder would cause. No existing treatment has been able to reach such results [8].

Psychological and/or educational interventions have proved their effectiveness on preventing anxiety disorders in a recent meta-analysis [9].

When it comes to physical activity, its implementation as a prevention program requires less economic resources and therefore results in a more cost-effective intervention, making it easier to implement at the first levels of sanitary assistance [10]. Exercise can be self-sustaining; patients can maintain it once the basic skills have been learnt [11]. Physical activity, defined as movement that is carried out by the skeletal muscles that results in energy expenditure [12], has been shown to provide health benefits for 26 chronic diseases [13], including anxiety [14,15,16]. A recent meta-analysis showed a small effect of exercise-based interventions on the reduction of depressive symptoms in people without clinical depression [17]. Practising physical activity also improves quality of life even in serious diseases such as cancer [18].

Several systematic reviews and meta-analyses have addressed the effects of physical activity in the prevention of anxiety. Overall, the results of these studies showed a positive effect of physical activity in the prevention of anxiety. However, some of these studies have not been limited to randomized controlled trials (RCTs), the designs that provide the most evidence of causality [19], and included other types of designs (e.g., quasi-experimental, longitudinal) [20,21,22]. Other studies included participants with and without anxiety, therefore, the effectiveness of prevention and the effectiveness of treatment could not be differentiated [14,21,23]. In all of these studies, anxiety was defined as any type of anxiety disorder or any type of anxiety symptoms measured by standardized interviews or validated anxiety symptoms scales.

This paper aims to evaluate the effectiveness of physical activity in the primary prevention of anxiety through a systematic review and meta-analysis of randomised controlled trials. Our specific research question is as follows: are physical activity-based interventions effective in reducing new cases of anxiety or anxiety symptoms in people without clinical anxiety?

## 2. Materials and Methods

To conduct this systematic review and meta-analysis, preferred reporting items for systematic review and meta-analysis (PRISMA) were followed [24]. We registered the protocol of this study at the International Prospective Register of Systematic Reviews (CRD42018094213).

### 2.1. Search Strategies

We systematically searched seven electronic databases, including MEDLINE (through PubMed), PsycINFO, Web of Science, EMBASE, SPORTDiscus, Cochrane Central Register of Controlled Trials (CENTRAL) and OpenGrey (System for Information on Grey Literature in Europe) from inception to 24 December 2021. No date or language restrictions were imposed. The reference lists of 56 reviews published on this topic were reviewed (see Appendix A). We also reviewed the reference lists of the studies selected. In addition, experts in the field were contacted and asked to complete the list of selected publications. Databases were searched separately by three of us. Search terms included: “physical activity”, “randomized controlled trial”, “anxiety disorder”, “intervention”, and “prevention”. To increase search sensitivity, search terms were used in their broadest sense. The search strategy was designed based on a preliminary search on PubMed and improved for further searching in other databases. The specific search strategies used are described in Appendix B.

### 2.2. Eligibility Criteria

We selected randomized clinical trials (RCTs) since they are the standard for clinical trials [25]. We focused on physical activity interventions, defined as “any bodily movement produced by skeletal muscles that results in energy expenditure above resting level”, which is planned, structured, and repetitive and can be characterized by type, intensity, frequency and time [12].

We excluded the RCTs which met any of the following exclusion criteria: (1) multicomponent intervention (e.g., physical activity + nutritional supplement), as long as they included another active component in the prevention of anxiety in addition to physical activity, without another arm of intervention consisting of exclusive physical activity; (2) no objective (e.g., recording devices, accelerometers) or subjective (e.g., validated questionnaires) verification of the performance of physical activity; (3) single control group consisting of an intervention already proven effective for the prevention of anxiety; and (4) not discarding anxiety at study baseline, either with standardized interviews (e.g., CIDI) or validated symptom scales with cut-off points (e.g., HADS-A), in order to separate the effectiveness of prevention from that to therapies.

The comparators allowed were care-as-usual, no intervention, a waiting list for intervention, or attention control.

Any type of anxiety disorder measured through standardized interviews or validated anxiety symptoms scales using standard cut-offs and/or any type of anxiety symptoms (physiological, cognitive or behavioural) measured through validated anxiety symptoms scale were eligible as outcomes. Participants could have any socio-demographic (e.g., age, sex, level of education) or clinical (e.g., healthy, chronic physical conditions) characteristic, and all settings and languages were considered.

### 2.3. Selection of Studies

To determine study eligibility, titles and abstracts and full-text studies were independently screened by three of us, and discrepancies were resolved by consensus, consistent with the process outlined for study eligibility.

### 2.4. Data Extraction

Data extraction was performed independently and in duplicate by three reviewers using an evidence table. Disagreements were resolved through consensus. Data extracted from each study included: author(s), year of publication, country, target population, type of prevention, baseline anxiety as exclusion criteria, sample size (control and intervention), characteristics of the physical activity program (frequency, intensity and type) and the control group; supervised or non-supervised physical activity, verification of physical activity (objective or subjective), type of outcome on anxiety (main or secondary), outcome measure instrument (standardized interview or symptom scale) and follow-up time. We contacted the authors of the original studies in case there was relevant information that was not reported in the studies.

### 2.5. Risk of Bias

We used the Cochrane Collaboration risk of bias tool to assess the quality of the studies included, based on the following criteria: random sequence generation, which refers to the rule used for allocating interventions to participants based on a random process; allocation concealment, which refers to the method employed to conceal the allocation sequence to determine whether interventions allocations could have been anticipated before or during enrolment; blinding of participants and personnel, which refers to the measures used to blind participants and study staff to the intervention each participant received; blinding of outcome assessment, which refers to the measures taken to blind the evaluators of outcomes to the intervention each participant received; completeness of outcome data, incomplete results data, refers to the methods used to deal with missing data (e.g., multiple imputation); and selective reporting, which refers to the availability of the trial protocol. They were rated as “high risk”, “unclear risk”, or “low risk” [26]. Studies that scored high risk of bias in specific domains (generation of the sequence, allocation concealment or blinding of evaluators of outcomes) were considered to have a high overall risk of bias. All of the studies included were assessed by three of us, and any disagreements were resolved by discussion.

### 2.6. Statistical Analysis

Since all outcomes of the included studies consisted of differences in anxiety symptoms between intervention and control groups, sample sizes, means, and standard deviations (SDs) were extracted for each RCT arm. Standardized mean differences (SMDs) were chosen as measure of effect and were therefore calculated for each study by estimating the mean SMD at different follow-up times. We then estimated the pooled SMDs for all studies and their 95%CIs. An improvement in the reduction of anxiety symptoms in the intervention group were represented by negative SMDs. Cohen suggested that an SMD of −0.2 is indicating a low effect; −0.5, a moderate effect; and −0.8, a large effect [27].

Fixed- and random-effect models were used to calculate the pooled SMDs and its CI. We selected the fixed effects model for the study under the assumption that the RCTs included in the meta-analysis were performed in a variety of populations that may not differ greatly from each other [26].

Standard errors of the nested comparisons in the same RCT were inflated following the Cates’ suggestions [28].

The I^2^ statistic describes the percentage of variability in effect estimates caused by heterogeneity rather than by sampling errors (chance). A value of 0% to 40% might indicate no important heterogeneity; 30% to 60%, moderate; 50% to 90%, substantial; and 75% to 100%, considerable [26]. The Q statistic and its *p* value were also calculated. For the introduction and analysis of data, version 2.2.064 of the Comprehensive Meta-Analysis statistical program was used (Biostat, Englewood, NJ).

### 2.7. Quality of Evidence

We assessed the quality of evidence using the grading of recommendations assessment, development and evaluation (GRADE) working group methodology [29]. We were taken into account the domains of risk of bias, consistency, directness, precision and publication bias.

## 3. Results

### 3.1. Search Results

**Figure 1:**
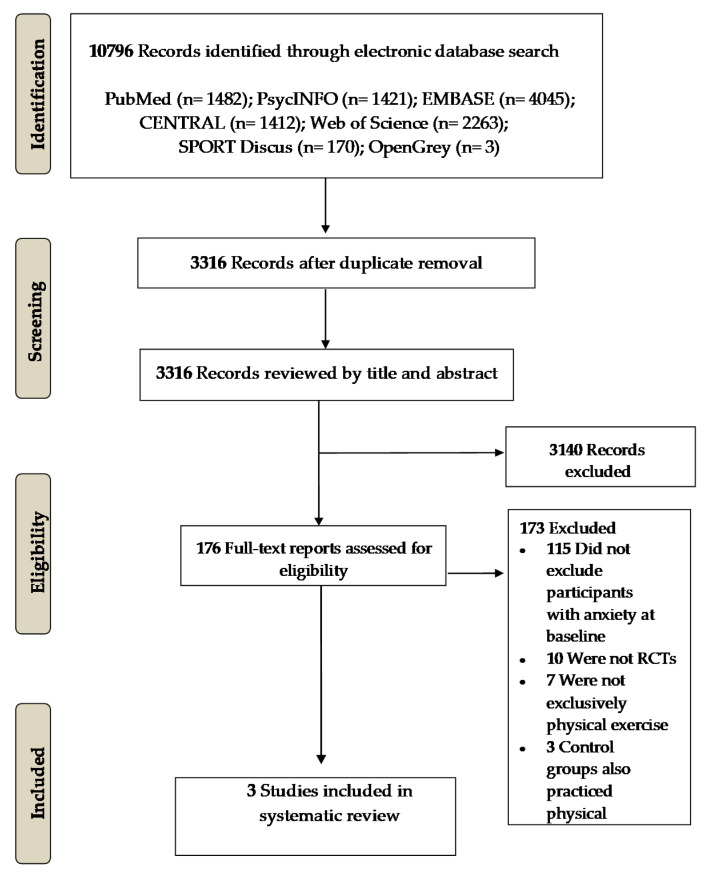
PRISMA flowchart of the randomized controlled trials included.

As shown in Figure 1, there were 10,796 studies found in the initial search, from which 7480 duplicated studies were removed. A total of 176 studies were reviewed in full-text and, finally, 3 RCTs (comprising 5 comparisons) were included in this systematic review and meta-analysis [30,31,32].

### 3.2. Characteristics of Included Studies

The characteristics of the 3 RCTs included are described in Table 1.

A total of 350 patients were enrolled (212 in the intervention group and 128 in the control group). The RCTs publication dates ranged from 2005 to 2015. The RCT locations were Taiwan [30], Canada [31], and Australia [32]. All RCTs were performed on adults.

Two RCTs evaluated selective prevention, one in women after breast cancer surgery [32] and the other on primary lung cancer patients [30]. Only one RCT evaluated universal prevention on healthy sedentary volunteers [31].

Two RCTs based their interventions in aerobic exercise programs consisting on walking at moderate intensity (60–80% max HR) [30,31] One RCT built its intervention program on an aerobic base, adding anaerobic exercise through time [32]. Exercise sessions had a duration between 20 to 45 min, three to five times per week.

One study assessed the effects of walking 30 min at a moderate intensity in two different settings: one daily session of continuous 30 min walking against three daily sessions of 10 min walking, separated from each other by a rest period of at least 2 h [31].

Another study designed its intervention so that exercise could be delivered face to face or via telephone, existing therefore two interventions arms [32].

Exercise sessions were not supervised in any of the three RCTs [30,31,32].

Nevertheless, study adherence was objectively verified in every RCT, using HR monitors and training diaries in two of them [30,31]. The other study verified the exercise adherence using the 3-min step modified test (6 inches instead of 12 inches, metronome set at 96 beats/min) and anaerobic strength tests (incremental exercise protocol combining a traditional upright row and shoulder press exercise using hand weights—ranging from 0 to 3.5 kg) as well as standardized surveys [32]. One RCT also measured perceived exertion using Borg’s rating scale [30]. One RCT assessed VO_2_Max values while performing the Balke and Ware treadmill test and percent body fat at baseline and follow-up [31].

The comparator used was care-as-usual in all of the 3 RCTs.

Follow-up periods ranged from 2 to 10 months (median 6 months).

Regarding the outcomes, all RCTs measured the reduction of anxiety symptoms using different scales.

### 3.3. Risk of Bias in Included Studies

The risk of bias for each study is reported in Table 2. For random sequence generation, we categorized two RCTs as being at low risk of bias, as they reported methods which consisted of computer randomization list [30,32], while one RCT did not provide information to make a judgement [31]. Two RCTs were judged as being at low risk of allocation concealment bias as they reported the use of sealed, opaque envelopes to conceal the allocation [30] and a computer program [32]. The other one did not report on allocation concealment so was judged as having high risk of bias [31]. The blinding of participants and personnel item was considered to be at high risk in all of the RCTs since it is extremely difficult to blind participants and personnel due to the nature of the intervention (physical activity). All of the three RCTs provided information on the blinding of outcome assessors and were rated as at low risk of bias. An intention-to-treat analysis and a method to impute missing data was undertaken in two RCTs [30,32]. The remaining RCT did not provide information about the imputation of missing data so were judged as having high risk of bias [31]. There was low risk of selective reporting bias in two RCTs as they provided the protocol trial registration [30,32]. No information about study protocol was available in the study carried out by Osei-Tutu et al. [31], so we judged it to be at high risk of bias.

### 3.4. Effectiveness of the Intervention to Prevent Anxiety

The study by Chen et al., 2015 [30] was aimed to determine the effectiveness of a walking exercise program in managing anxiety, depression and the severity of cancer-related symptoms in Taiwanese patients with lung cancer. The program consisted of doing moderate intensity home-based walking-exercise, 3 times per week, and weekly exercise counselling during 12 weeks. This program was compared with usual care. There were no statistically significant differences in anxiety scores between groups at the end of the follow-up in patients who scored less than 8 points in HAD-A at baseline.

Osei-Tutu et al., 2005 [31] performed a study with sedentary Canadian subjects to determine the effects of short and long bouts of physical activity compared with usual care on mood. Both the short bouts and long bouts consisted of walking five days per week during eight weeks. On the one hand, in the short bouts, participants had to accumulate 30 min of walking in three, 10 min sessions separated by intervals of more than 2 h. On the other hand, in the long bouts, participants had to perform a single 30 min continuous walking. In non-anxious subjects at baseline, the results showed that anxiety decreased significantly only in the long bouts of physical activity.

The purpose of the study conducted by Hayes et al., 2012 [32] was to evaluate the effectiveness of an exercise for health intervention delivered either face to face or telephone, compared with usual care in Australian women with post-breast cancer surgery on anxiety and quality of life among others. The exercise intervention included aerobic and strength-based exercises four times per week during eight months. All sessions were tailored and progressively increasing the intensity from low to high. In non-anxious subjects at baseline, the exercise intervention did not seem to affect rates of anxiety at the end of the follow-up.

**Figure 2:**
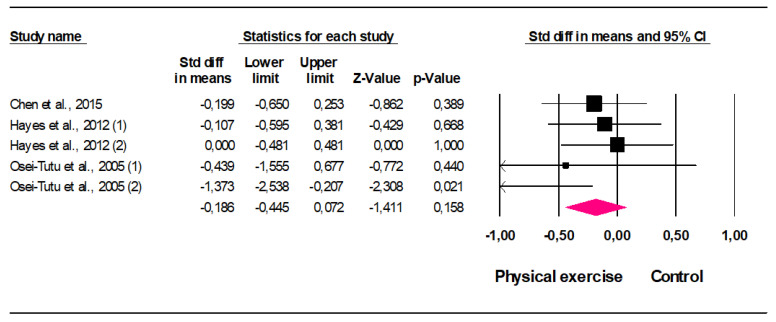
Forest Plot (fixed effects).

Five comparisons from three studies were used to perform meta-analysis calculations. The resulting forest plot for the overall and individual sizes can be reviewed in Figure 2. The pooled SMD assuming the fixed-effects model was −0.18 (95% CI: −0.45 to 0.07; *p* = 0.158) and the equivalent pooled odds ratio (OR) was 0.71 (95% CI: 0.44 to 1.14; *p* = 0.158). The heterogeneity was irrelevant (I^2^ = 17,67%) and not statistically significant (Q = 4.859; d.f. = 4; *p* = 0.3), meaning that the effect sizes are homogeneous and, consequently, the pooled effect size represent them well [33]. This finding means that physical activity may have a small effect on anxiety prevention, but the lack of statistical power does not allow to make any recommendations. When estimates were made by the random-effects model, the result was similar (pooled SMD: −0.21; 95% CI: −0.50 to 0.09; *p* = 0.176).

### 3.5. Quality of the Evidence

According GRADE, the quality of evidence was very low. The starting point for GRADE was high since we included only RCT. The possible publication bias could not be estimated therefore we reduced the rating from high to moderate. We once again downgraded from moderate to low since only three trials were included (imprecision). The risk of bias was high since one of the studies scored high in the domains that we previously considered. Therefore, we once again reduced the rating from low to very low. RCT included tested physical exercise interventions in head-to-head comparisons. We did not find inconsistency since the outcome effects were consistent across RCT. Finally, the target population, the interventions, and our outcome did not differ from those of primary interest therefore indirectness was low.

## 4. Discussion

### 4.1. Summary

We have not found evidence that anxiety can be prevented through physical activity. Most of RCTs published did not excluded participants with anxiety at baseline and due to this only three of them were included in this systematic review. The three studies were conducted in three and two different countries and continents, respectively. One RCT was implemented in healthy sedentary volunteers, and the others in patients with cancer. The sample sizes were small, their follow-up short, and the risk of biases variable.

### 4.2. Strenghts

As far as we know, this is the first meta-analysis to evaluate the effectiveness of physical activity in the prevention of anxiety. In our study, we used a large number of electronic databases that were complementary (biomedical, psychosocial, grey literature, and specific for RCTs and sport). Search was complemented with manual search of reference lists of other systematic reviews and meta-analyses. In addition, the search terms used were wide enough for the search to have an adequate sensitivity. The selection of studies, evaluation of the risk of bias, and extraction of data from trials were performed by three independent and trained evaluators who solved the discrepancies by consensus. Another strength of our study is that only RCTs were included in our systematic review, since they are the designs that provide more evidence of causality. In addition, analyzing only RCTs with a study population free of anxiety at baseline allowed us to distinguish prevention from treatment effectiveness.

### 4.3. Limitations

The main limitation of our study is that only three RCTs met our inclusion criteria. Furthermore, the external validity is limited since together it only represents 350 people, most of them with cancer. In any case, when a meta-analysis is approached from the fixed effects model (as it happens to be in our study) it makes sense to estimate a combined effect size from just two RCTs [34]. For drawing conclusions about the existence of publication bias, it has been suggested that a meta-analysis requires a minimum of 10 studies [35].

The duration of the follow-up did not exceed 12 months in any of the 3 RCTs, and so conclusions about long-term effectiveness cannot be drawn from our study.

Due to the lack of greater available literature, we were not able to perform subgroup analyses in order to identify, for example, any specific type of exercise with greater effect on anxiety prevention or other differences concerning some socio-demographic (e.g., stretches of ages), clinical (e.g., healthy vs. physical chronic), or methodological (e.g., short-term vs. long-term follow-up) characteristics.

Regarding the outcomes, reduction of anxiety symptoms (measured by symptoms scales) was the only outcome used to measure the effect of the intervention. Although the reliability and validity of scales are widely accepted, standardized diagnostic interviews generally have greater validity. Nevertheless, the reduction of anxiety symptoms is also useful as an outcome since it has a positive and relevant effect on quality of life and cost [36,37]. In addition, since all studies assessed anxiety symptoms, we were not able to determine the effect of physical activity on the different anxiety responses.

### 4.4. Comparison with Existing Literature

Our meta-analysis did not find effectiveness of physical activity in the prevention of anxiety. A meta-analysis on anxiety prevention reports a small and not statistically significant SMDs [18], and another one on the effectiveness of tai-chi only calculated pooled SMDs for depression but not for anxiety [21]. However, participants with baseline anxiety were not excluded in any of these two studies. Therefore, their findings are not applicable to the prevention of anxiety. In any case, their inferences could be made in relation to the set of participants with and without anxiety. Other meta-analyses on the effectiveness of physical activity in preventing depression also have the same limitation [18,23,37,38,39]. However, recently, our research group has published a meta-analysis where depressed people at baseline were discarded, showing a small effect of exercise-based interventions on the reduction of depressive symptoms [17].

It should be noted that, in contrast to our findings, two meta-analyses [20,22] conducted in non-anxious clinical populations found that physical activity had a small but significant reductive effect on anxiety. However, these studies did not focus on RCTs, the design that provides more evidence on causality [19]. The effect of physical activity on anxiety prevention might be associated with the type of anxiety response. Therefore, further studies are needed to clarify the role of physical activity in different manifestations of anxiety.

A higher number of meta-analyses has been released in relation to the treatment of anxiety disorders with physical activity rather than its prevention [21,23,40,41,42,43]. These studies conclude that exercise may be a useful treatment for anxiety, but lack of data from rigorous, methodologically sound RCTs precludes any definitive conclusions about its effectiveness.

Although the exact pathways by which exercise may prevent and/or reduce anxiety are not known; social, psychological, and biological mechanisms may be involved [16]. Some research has proposed its relationship with the increase of ß-endorphins, endocannabinoids, monoamines and BDNF [44,45]. Physical activity has also been proven to produce plasticity in neural circuitry that results in DRN 5-HT neuronal constraint and resistance against the behavioral impact of acute increases in 5-HT. Evidence suggests that plasticity at multiple sites within the central 5-HT system converges to facilitate stress resistance and resilience [46].

## 5. Conclusions

Our study clearly showed lack of evidence on the effectiveness of physical activity for the primary prevention of anxiety. Specifically, this review found no evidence for aerobic exercise consisting of moderate-intensity walking or aerobic exercise with the addition of anaerobic exercise on the reduction of anxiety symptoms in people without clinical anxiety. Larger, long-term RCTs with low risk of bias, where baseline anxiety is discarded and standardized structured interviews are employed to evaluate the occurrence of anxiety are needed. Currently, more evidence is needed in order to make recommendations on the effect of physical activity in the prevention of anxiety.

## Figures and Tables

**Table 1:** Characteristics of randomized controlled trials included.

Author—Year—Country	Target Population—Type of Prevention	Anxiety Exclusion at Baseline	Sample (Intervention-Control)	Conditions (Intervention–Control)	Characteristics of Physical Activity [(a) Frequency, (b) Intensity, (c) Type, (d) Supervised/Unsupervised]	Verification of Physical Activity (Objective—Subjective)	Type of Outcome on Anxiety (Main—Secondary)	Follow-Up (Months)	Anxiety Outcomes (Standardized Interview—Symptoms Scale)
Chen et al., 2015 Taiwan	Primary lung cancer patients 37–88 years Mean age 64.16 ± 10.89 years Selective	No anxiety (HADS-A < 8) ^1^	95(45/50) ^2^	Walking Usual care	(a) 40 min × 3 times per week × 12 weeks (b) Moderate (60–80% max HR) (c) Walking aerobic (d) Unsupervised	Objective: Heart rate monitor + training diary Subjective: Borg rating of perceived exertion scale	Main	6 months	Symptom’s scale (HADS-A) ^1^
Osei-Tutu et al., 2005 Canada	Healthy sedentary volunteers 20–40 years Universal	No anxiety (POMS) ^3^	40 (9/11/10)	30′ walking 3 × 10′ walking with minimum 2 h rest intervals Usual care	(a) 30 min × 5 times per week × 8 weeks (b) Moderate (60–80% max HR) (c) Walking aerobic (d) Unsupervised	Objective: Heart rate monitor + training diary + VO_2_Max + percent body fat	Main	2 months	Symptom’s scale (POMS) ^2^
Hayes et al., 2012 Australia	Women after breast cancer surgery 20–69 years Selective	No anxiety (Greene Climacteric scale, Anxiety subscale ≤ 9)	194 (67/67/60)	Face to face: > 180 min aerobic and/or strength exercise per week Telephone: > 180 min aerobic and/or strength exercise per week Usual care	(a) Progressive (> 4 sessions of 20 min per week in weeks 1–4; 30 min weeks 5–8; 45 min weeks 8–32) total duration 8 months. (b) Progressive (low weeks 1–4; moderate weeks 5–8; moderate-high weeks 8–32) (c) Combined (aerobic weeks 1–4, aerobic + strength weeks 5–32) (d) Unsupervised	Objective: Heart rate during 3 min modified step test + incremental exercise protocol combining a traditional upright row and shoulder press exercise using hand weights + Active Australia Survey	Main	10 months	Symptom’s scale (Greene Climacteric scale, Anxiety subscale)

^1^ HADS-A, Hospital Anxiety and Depression Scale—Anxiety Subscale; ^2^ Data on non-anxious people were provided by the authors; ^3^ POMS, Profile of Mood States.

**Table 2:** Risk of bias of randomized controlled trials included to prevent anxiety.

Study	Selection Bias		Performance Bias	Detection Bias	Attrition Bias	Reporting Bias
	Random Sequence Generation	Allocation Concealment	Blinding of Participants and Personnel	Blinding of Outcome Assessment	Incomplete Outcome Data	Selective Reporting
Chen et al., 2015	Low risk	Low risk	High risk	Low risk	Low risk	Low risk
Osei-Tutu et al., 2005	High risk	High risk	High risk	Low risk	High risk	High risk
Hayes et al., 2012	Low risk	Low risk	High risk	Low risk	Low risk	Low risk

## Data Availability

Not applicable.

## Appendix A

References of 56 Reviewed Systematic Reviews and Meta-analyses.

### References of 56 Reviewed Systematic Reviews and Meta-Analyses

1. Brown HE, Gilson ND, Burton NW, Brown WJ. Does physical activity impact on presenteeism and other indicators of workplace well-being? Sports Med. 2011;41(3):249-262. doi:10.2165/11539180-000000000-00000.
2. Potter R, Ellard D, Rees K, Thorogood M. A systematic review of the effects of physical activity on physical functioning, quality of life and depression in older people with dementia. Int J Geriatr Psychiatry. 2011;26(10):1000-1011. doi:10.1002/gps.2641.
3. Das JK, Salam RA, Lassi ZS, et al. Interventions for adolescent mental health: an overview of systematic reviews. J Adolesc Health. 2016;59(4S):S49-S60. doi:10.1016/j.jadohealth.2016.06.020.
4. Wegner M, Helmich I, Machado S, Nardi AE, Arias-Carrion O, Budde H. Effects of exercise on anxiety and depression disorders: review of meta-analyses and neurobiological mechanisms. CNS Neurol Disord Drug Targets. 2014;13(6):1002-1014.
5. Rosenbaum S, Vancampfort D, Steel Z, Newby J, Ward PB, Stubbs B. Physical activity in the treatment of Post-traumatic stress disorder: a systematic review and meta-analysis. Psychiatry Res. 2015;230(2):130-136. doi:10.1016/j.psychres.2015.10.017.
6. Hall KS, Hoerster KD, Yancy WS. Post-traumatic stress disorder, physical activity, and eating behaviors. Epidemiol Rev. 2015;37:103-115. doi:10.1093/epirev/mxu011.
7. Mammen G, Faulkner G. Physical activity and the prevention of depression: a systematic review of prospective studies. Am J Prev Med. 2013;45(5):649-657. doi:10.1016/j.amepre.2013.08.001.
8. Zhai L, Zhang Y, Zhang D. Sedentary behaviour and the risk of depression: a meta-analysis. Br J Sports Med. 2015;49(11):705-709. doi:10.1136/bjsports-2014-093613.
9. Park SH, Han KS, Kang CB. Effects of exercise programs on depressive symptoms, quality of life, and self-esteem in older people: a systematic review of randomized controlled trials. Appl Nurs Res. 2014;27(4):219-226. doi:10.1016/j.apnr.2014.01.004.
10. Cooney GM, Dwan K, Greig CA, et al. Exercise for depression. Cochrane Database Syst Rev. 2013;(9):Cd004366. doi:10.1002/14651858.CD004366.pub6.
11. Cramer H, Lauche R, Langhorst J, Dobos G. Yoga for depression: a systematic review and meta-analysis. Depress Anxiety. 2013;30(11):1068-1083. doi:10.1002/da.22166.
12. Robertson R, Robertson A, Jepson R, Maxwell M. Walking for depression or depressive symptoms: a systematic review and meta-analysis. Ment Health Phys Act. 2012;5(1):66-75.
13. de Souza Moura AM, Lamego MK, Paes F, et al. Effects of aerobic exercise on anxiety disorders: a systematic review. CNS Neurol Disord Drug Targets. 2015;14(9):1184-1193. doi:10.2174/1871527315666151111121259.
14. Nystrom MB, Neely G, Hassmen P, Carlbring P. Treating major depression with physical activity: a systematic overview with recommendations. Cogn Behav Ther. 2015;44(4):341-352. doi:10.1080/16506073.2015.1015440.
15. Rebar AL, Stanton R, Geard D, Short C, Duncan MJ, Vandelanotte C. A meta-meta-analysis of the effect of physical activity on depression and anxiety in non-clinical adult populations. Health Psychol Rev. 2015;9(3):366-378. doi:10.1080/17437199.2015.1022901.
16. Yan S, Jin Y, Oh Y, Choi Y. Effect of exercise on depression in university students: a meta-analysis of randomized controlled trials. J Sports Med Phys Fitness. 2016;56(6):811-816.
17. Josefsson T, Lindwall M, Archer T. Physical exercise intervention in depressive disorders: meta-analysis and systematic review. Scand J Med Sci Sports. 2014;24(2):259-272. doi:10.1111/sms.12050.
18. Schuch FB, Vancampfort D, Richards J, Rosenbaum S, Ward PB, Stubbs B. Exercise as a treatment for depression: a meta-analysis adjusting for publication bias. J Psychiatr Res. 2016b;77:42-51. doi:10.1016/j.jpsychires.2016.02.023.
19. Farah, WH, Alsawas, M, Mainou, M, et al. Non-pharmacological treatment of depression: a systematic review and evidence map. Evid Based Med. 2016;21(6):214-221.
20. Mura G, Carta MG. Physical activity in depressed elderly. A systematic review. Clin Pract Epidemiol Ment Health. 2013;9:125-135. doi:10.2174/1745017901309010125.
21. Schuch FB, Vancampfort D, Rosenbaum S, et al. Exercise for depression in older adults: a meta-analysis of randomized controlled trials adjusting for publication bias. Rev Bras Psiquiatr. 2016c;38(3):247-254. doi:10.1590/1516-4446-2016-1915.
22. Rhyner KT, Watts A. Exercise and depressive symptoms in older adults: a systematic meta-analytic review. J Aging Phys Act. 2016;24(2):234-246. doi:10.1123/japa.2015-0146.
23. Lindheimer JB, O’Connor PJ, Dishman RK. Quantifying the placebo effect in psychological outcomes of exercise training: a meta-analysis of randomized trials. Sports Med. 2015;45(5):693-711. doi:10.1007/s40279-015-0303-1.
24. Cramer H, Anheyer D, Lauche R, Dobos G. A systematic review of yoga for major depressive disorder. J Affect Disord. 2017;213:70-77. doi:10.1016/j.jad.2017.02.006.
25. Bridges L, Sharma M. The efficacy of yoga as a form of treatment for depression. J Evid Based Complementary Altern Med. 2017;2156587217715927. doi:10.1177/2156587217715927.
26. Liu X, Clark J, Siskind D, et al. A systematic review and meta-analysis of the effects of Qigong and Tai Chi for depressive symptoms. Complement Ther Med. 2015;23(4):516-534. doi:10.1016/j.ctim.2015.05.001.
27. Sarris J, Moylan S, Camfield DA, et al. Complementary medicine, exercise, meditation, diet, and lifestyle modification for anxiety disorders: a review of current evidence. Evid Based Complement Alternat Med. 2012. 2012:809653. doi:10.1155/2012/809653.
28. Yin J, Dishman RK. The effect of Tai Chi and Qigong practice on depression and anxiety symptoms: a systematic review and meta-regression analysis of randomized controlled trials. Database of Abstracts of Reviews of Effects. 2014;(2):135-146.
29. Wang F, Lee Ek, Wu T, et al. The effects of Tai Chi on depression, anxiety, and psychological well-being: a systematic review and meta-analysis. Int J Behav Med. 2014;21(4):605-617.
30. Meekums B, Karkou V, Nelson EA. Dance movement therapy for depression. Cochrane Database Syst Rev. 2015;(2):CD009895. doi:10.1002/14651858.CD009895.pub2.
31. Loi SM, Dow B, Ames D, et al. Physical activity in caregivers: What are the psychological benefits? Arch Gerontol Geriatr. 2014;59(2):204-210. doi:10.1016/j.archger.2014.04.001.
32. Abraha I, Rimland JM, Trotta FM, et al. Systematic review of systematic reviews of non-pharmacological interventions to treat behavioural disturbances in older patients with dementia. The SENATOR-OnTop series. BMJ Open. 2017;7(3):e012759. doi:10.1136/bmjopen-2016-012759.
33. Barreto Pde S, Demougeot L, Pillard F, Lapeyre-Mestre M, Rolland Y. Exercise training for managing behavioral and psychological symptoms in people with dementia: A systematic review and meta-analysis. Ageing Res Rev. 2015;24(Pt B):274-285. doi:10.1016/j.arr.2015.09.001.
34. Adamson BC, Ensari I, Motl RW. Effect of exercise on depressive symptoms in adults with neurologic disorders: a systematic review and meta-analysis. Arch Phys Med Rehabil. 2015;96(7):1329-1338. doi:10.1016/j.apmr.2015.01.005.
35. Eng JJ, Reime B. Exercise for depressive symptoms in stroke patients: a systematic review and meta-analysis. Clin Rehabil. 2014;28(8):731-739. doi:10.1177/0269215514523631.
36. Radovic S, Gordon MS, Melvin GA. Should we recommend exercise to adolescents with depressive symptoms? A meta-analysis. J Paediatr Child Health. 2017;53(3):214-220. doi:10.1111/jpc.13426.
37. Brown H, Pearson N, Braithwaite R, Brown W, Biddle S. Physical activity interventions and depression in children and adolescents: a systematic review and meta-analysis. Sports Med. 2013;43:195-206. doi:10.1007/s40279-012-0015-8.
38. Hoare E, Skouteris H, Fuller-Tyszkiewicz M, Millar L, Allender S. Associations between obesogenic risk factors and depression among adolescents: a systematic review. Obes Rev. 2014;15(1):40-51. doi:10.1111/obr.12069.
39. Hoare E, Milton K, Foster C, Allender S. The associations between sedentary behaviour and mental health among adolescents: a systematic review. Int J Behav Nutr Phys Act. 2016;13(1):108. doi:10.1186/s12966-016-0432-4.
40. Korczak DJ, Madigan S, Colasanto M. Children’s physical activity and depression: a meta-analysis. Pediatrics. 2017;139(4):1-14.
41. Carter T, Morres ID, Meade O, Callaghan P. The effect of exercise on depressive symptoms in adolescents: a systematic review and meta-analysis. J Am Acad Child Adolesc Psychiatry. 2016;55(7):580-590. doi:10.1016/j.jaac.2016.04.016.
42. Schuch FB, Deslandes AC, Stubbs B, Gosmann NP, Silva CT, Fleck MP. Neurobiological effects of exercise on major depressive disorder: a systematic review. Neurosci Biobehav Rev. 2016a;61:1-11. doi:10.1016/j.neubiorev.2015.11.012.
43. Pedersen BK, Saltin B. Exercise as medicine – evidence for prescribing exercise as therapy in 26 different chronic diseases. Scand J Med Sci Sports 2015: (Suppl. 3) 25: 1–72.
44. Knapen J, Vancampfort D, Moriën Y, Marchal Y. Exercise therapy improves both mental and physical health in patients with major depression. Disabil Rehabil. 2015;37(16):1490-5.
45. Stubbs B, Vancampfort D, Rosenbaum S, Ward PB, Richards J, Ussher M, Schuch FB. Challenges Establishing the Efficacy of Exercise as an Antidepressant Treatment: A Systematic Review and Meta-Analysis of Control Group Responses in Exercise Randomised Controlled Trials. Sports Med. 2016;46(5):699-713.
46. Herring MP, Puetz TW, O’Connor PJ, Dishman RK. Effect of exercise training on depressive symptoms among patients with a chronic illness: a systematic review and meta-analysis of randomized controlled trials. Arch Intern Med. 2012;172(2):101-11.
47. Conn VS. Depressive symptom outcomes of physical activity interventions: meta-analysis findings. Ann Behav Med. 2010;39(2):128-38.
48. Ekeland E, Heian F, Hagen KB. Can exercise improve self-esteem in children and young people? A systematic review of randomised controlled trials. Br J Sports Med 2005;39(11):792-8.
49. Carter T, Bastounis A, Guo B, Morrell CJ. The effectiveness of exercise-based interventions for preventing or treating postpartum depression: a systematic review and meta-analysis. Archives of Women’s Mental Health 2019;22(1): 37-53
50. Chan JSY, Liu G, Liang D, Deng K, Wu J, Yan JH. Special Issue- Therapeutic Benefits of Physical Activity for Mood: A systematic review on the Effects of Exercise Intensity, Duration and Modality. The Journal of Psychology 2019;153(1):102-125.
51. Nakamura A, van der Waerden J, Melchior M, Bolze C, El-Khoury F, Pryor L. Physical activity during pregnancy and postpartum depression: A systematic review and meta-analysis. Journal of Affective Disorders 2019;246:29-41
52. Pascoe M C, Parker AG. Physical activity and exercise as an universal depression prevention in young people: a narrative review. Early Interv Psychiatry. 2019;13(4):733-739
53. Davenport MH, McCurdy AP, Mottola MF, Skow RJ, Meah VL, Poitras VJ, Jaramillo Garcia A, Gray CE, Barrowman N, Riske L, Sobierajski F, James M, Nagpal T, Marchand AA, Nuspl M, Slater LG, Barakat R, Adamo KB, Davies GA, Ruchat SM. Impact of prenatal exercise on both prenatal and postnatal anxiety and depressive symptoms: a systematic review and meta-analysis.Br J Sports Med. 2018 Nov;52(21):1376-1385
54. O’Connor E, Senger CA, Henninger ML, Coppola E, Gaynes BN. Interventions to Prevent Perinatal Depression: Evidence Report and Systematic Review for the US Preventive Services Task Force. JAMA. 2019;321(6):588-601.
55. Gordon BR, McDowell CP, Hallgren M, Meyer JD, Lyons M, HerringMP. Association of efficacy of resistance exercise training with depressive symptoms. JAMA Psychiatry. 2018;75(6):566-576.
56. Poyatos-León R, García-Hermoso A, Sanabria-Martínez G, Álvarez-Bueno C, Cavero-Redondo I, Martínez-Vizcaíno V. Effects of exercise-based interventions on postpartum depression: A meta-analysis of randomized controlled trials. Birth. 2017 Sep;44(3):200-208.

## Appendix B

Electronic search strategies.

### Electronic Search Strategies

- **PubMed (MEDLINE):**

(“anxiety disorders”[MeSH Terms] OR (“anxiety”[All Fields] AND “disorders”[All Fields]) OR “anxiety disorders”[All Fields] OR (“anxiety”[All Fields] AND “disorder”[All Fields]) OR “anxiety disorder”[All Fields] OR “anxiety”[MeSH Terms] OR “anxiety”[All Fields]) AND (“physical activity” OR “exercise” OR “fitness” OR “Sport” OR “leisure activities”) AND (prevent* OR incidence) AND (“effectiveness” OR “trial” OR “controlled trial” OR “randomi*” OR “intervention” OR “efficacy”)

- **PsycINFO:**

(anxiety OR anxiety disorders OR (anxiety AND disorder)) AND (physical activity OR exercise OR fitness OR Sport OR leisure activities) AND (prevent* OR incidence) AND (effectiveness OR trial OR (controlled AND trial) OR randomi* OR intervention OR efficacy)

- **Web of Science:**

TS = ((anxiety disorders OR (anxiety AND disorders) OR anxiety)) AND TS = ((physical activity OR exercise OR fitness OR Sport OR leisure activities)) AND TS = ((prevent* OR incidence)) AND TS = ((effectiveness OR trial OR (controlled AND trial) OR randomi* OR intervention OR eficacy))

- **EMBASE:**

(’anxiety’/exp OR ’anxiety’ OR ’anxiety disorder’/exp OR ’anxiety disorder’) AND (’physical activity’/exp OR ’physical activity’ OR ’exercise’/exp OR ’exercise’ OR ’fitness’/exp OR ’fitness’ OR ’Sport’/exp OR ’sport’ OR ’leisure activities’/exp OR ’leisure activities’) AND (prevent* OR incidence) AND (’effectiveness’ OR ’trial’ OR ’controlled trial’ OR ’randomi*’ OR ’intervention’ OR ’eficacy’)

- **SPORTDiscus:**

(“anxiety” OR “anxiety disorders” OR (anxiety AND disorder)) AND (“physical activity” OR “exercise” OR “fitness” OR “Sport” OR “leisure activities”) AND (prevent* OR incidence) AND (“effectiveness” OR “trial” OR “controlled trial” OR “randomi*” OR “intervention” OR “efficacy”)

- **Cochrane Central Register of Controlled Trials:**

(“anxiety” or “anxiety disorder”):ti,ab,kw AND (physical activity or exercise or fitness or Sport or leisure activities):ti,ab,kw AND (prevent* or incidence):ti,ab,kw AND (effectiveness or trial or controlled trial or randomi* or intervention or efficacy):ti,ab,kw

- **OpenGrey:**

(anxiety) AND (prevent*) AND (physical activity OR exercise OR fitness OR Sport OR leisure activities)

## References

[1] GBD 2019 Diseases and Injuries Collaborators (2020). Global burden of 369 diseases and injuries in 204 countries and territories, 1990–2019: A systematic analysis for the Global Burden of Disease Study 2019. Lancet.

[2] GBD 2017 Disease and Injury Incidence and Prevalence Collaborators (2018). Global, regional, and national incidence, prevalence, and years lived with disability for 354 diseases and injuries for 195 countries and territories, 1990–2017: A systematic analysis for the Global Burden of Disease Study 2017. Lancet.

[3] Walker E.R., McGee R.E., Druss B.G. (2015). Mortality in Mental Disorders and Global Disease Burden Implications. A Systematic Review and Meta-analysis. JAMA Psychiatry.

[4] Whiteford H.A., Degenhardt L., Rehm J., Baxter A.J., Ferrari A.J., Erskine H.E., Charlson F.J., Norman R.E., Flaxman A.D., Johns N. (2013). Global Burden of Disease Attributable to Mental and Substance Use Disorders: Findings from the Global Burden of Disease Study 2010. Lancet.

[5] Acarturk C., Cuijpers P., van Straten A., de Graaf R. (2009). Psychological treatment of social anxiety disorder: A meta-analysis. Psychol. Med..

[6] Cuijpers P., Sijbrandij M., Koole S., Huibers M., Berking M., Andersson G. (2014). Psychological treatment of generalized anxiety disorder: A meta-analysis. Clin. Psychol. Rev..

[7] Fernández A., Haro J.M., Martinez-Alonso M., Demyttenaere K., Brugha T.S., Autonell J., De Girolamo G., Bernert S., Lepine J.P., Alonso J. (2007). Treatment adequacy for anxiety and depressive disorders in six European countries. Br. J. Psychiatry.

[8] Andrews G., Issakidis C., Sanderson K., Corry J., Lapsley H. (2004). Utilising survey data to inform public policy: Comparison of the cost-effectiveness of treatment of ten mental disorders. Br. J. Psychiatry.

[9] Moreno-Peral P., Conejo-Cerón S., Rubio-Valera M., Fernández A., Navas-Campaña D., Rodríguez-Morejón A., Motrico E., Rigabert A., de Dios Luna J., Martín-Pérez C. (2017). Effectiveness of Psychological and/or Educational Interventions in the Prevention of Anxiety. A Systematic Review, Meta-analysis, and Meta-regression. JAMA Psychiatry.

[10] Laine J., Kuvaja-Köllner V., Pietilä E., Koivuneva M., Valtonen H., Kankaanpää E. (2014). Cost-effectiveness of population-level physical activity interventions: A systematic review. Am. J. Health Promot..

[11] Mercer T.H., Naish P.F., Gleeson N.P., Crawford C. (2002). Low volume exercise rehabilitation improves functional capacity and self-reported functional status of dialysis patients. Am. J. Phys. Med. Rehab..

[12] Caspersen C.J., Powell K.E., Christenson G.M. (1985). Physical activity, exercise, and physical fitness: Definitions and distinctions for health-related research. Public Health Rep..

[13] Pedersen B.K., Saltin B. (2015). Exercise as medicine-evidence for prescribing exercise as therapy in 26 different chronic diseases. Scand J. Med. Sci. Sports.

[14] Herring M.P., O’Connor P.J., Dishman R.K. (2010). The effect of exercise training on anxiety symptoms among patients: A systematic review. Arch. Intern. Med..

[15] Stich F.A. (1999). A Meta-Analysis of Physical Exercise as a Treatment for Symptoms of Anxiety and Depression.

[16] Salmon P. (2001). Effects of physical exercise on anxiety, depression, and sensitivity to stress. Clin. Psychol. Rev..

[17] Bellón J.Á., Conejo-Cerón S., Sánchez-Calderón A., Rodríguez-Martín B., Bellón D., Rodríguez-Sánchez E., Mendive J.M., Ara I., Moreno-Peral P. (2021). Effectiveness of exercise-based interventions in reducing depressive symptoms in people without clinical depression: Systematic review and meta-analysis of randomised controlled trials. Br. J. Psychiatry.

[18] Courneya K.S. (2001). Exercise interventions during cancer treatment: Biopsychosocial outcomes. Exerc. Sports Sci. Rev..

[19] Piantadosi S. (2017). Clinical Trials: A Methodologic Perspective.

[20] Conn V.S. (2010). Anxiety outcomes after physical activity interventions: Meta-analysis findings. Nurs. Res..

[21] Wang F., Lee E.K.O., Wu T., Benson H., Fricchione G. (2004). The effects of tai chi on depression, anxiety, and psychological well-being: A systematic review and meta-analysis. Int. J. Behav. Med..

[22] McDowell C.P., Dishman R.K., Gordon B.R., Herring M.P. (2019). Physical Activity and Anxiety: A Systematic Review and Meta-analysis of Prospective Cohort Studies. Am. J. Prev. Med..

[23] Larun L., Nordheim L.V., Ekeland E., Hagen K.B., Heian F. (2006). Exercise in prevention and treatment of anxiety and depression among children and young people. Cochrane Database Syst. Rev..

[24] Moher D., Liberati A., Tetzlaff J., Altman D.G., PRISMA Group (2009). Preferred reporting items for systematic reviews and meta-analyses: The PRISMA Statement. BMJ.

[25] Piantadosi S. (2005). Clinical Trials: A Methodological Perspective.

[26] Higgins J.P.T., Green S. (2018). Cochrane Handbook for Systematic Reviews of Interventions. Version 5.1.0. http://handbook.cochrane.org/.

[27] Cohen J.A. (1992). Power primer. Psychol. Bull..

[28] Cates C. (2018). Multiple-Arm Trial Data: Using a Corrected Standard Error for GIV Analyses. Cochrane Colloquium. https://abstracts.cochrane.org/2015-vienna/multiple-arm-trial-data-using-corrected-standard-error-giv-analyses.

[29] Balshem H., Helfand M., Schünemann H.J., Oxman A.D., Kunz R., Brozek J., Vist G.E., Falck-Ytter Y., Meerpohl J., Norris S. (2011). GRADE guidelines: 3. Rating the quality of evidence. J. Clin. Epidemiol..

[30] Chen H.M., Tsai C.M., Wu Y.C., Lin K.C., Lin C.C. (2015). Randomised controlled trial on the effectiveness of home-based walking exercise on anxiety, depression and cancer-related symptoms in patients with lung cancer. Br. J. Cancer.

[31] Osei-Tutu K.B., Campagna P.D. (2005). The effects of short- vs. long-bout exercise on mood, VO 2MAX and percent body fat. Prev Med. An. Int. J. Devoted Pract. Theory.

[32] Hayes S.C., Rye S., DiSipio T., Yates P., Bashford J., Pyke C., Saunders C., Battistutta D., Eakin E. (2013). Exercise for health: A randomized, controlled trial evaluating the impact of a pragmatic, translational exercise intervention on the quality of life, function and treatment-related side effects following breast cancer. Breast Cancer Res. Treat..

[33] Higgins J.P., Thomson S.G., Deeks J.J., Altman D.G. (2011). Measuring inconsistency in meta-analyses. BMJ.

[34] Borenstein M., Hedges L.V., Higgins J.P.T., Rothstein H.R., Borenstein M., Hedges L.V., Higgins J.P.T., Rothstein H.R. (2009). When does it make sense to perform a meta-analysis?. Introduction to Meta-Analysis.

[35] Harbord R.M., Egger R.J., Sterne J.A.C. (2006). A modified test for small study effects in meta-analysis of controlled trials with binary endpoint. Stat. Med..

[36] Abedi P., Nikkhah P., Najar S. (2015). Effect of pedometer-based walking on depression, anxiety and insomnia among postmenopausal women. Climacteric.

[37] Nelson D.B., Sammel M.D., Freeman E.W., Lin H., Gracia C.R. (2008). Effect of physical activity on menopausal symptoms among urban women. Med. Sci. Sports Exerc..

[38] Mammen G., Faulkner G. (2013). Physical activity and the prevention of depression: A systematic review of prospective studies. Prev. Med..

[39] Herring M.P., Puetz T.W., O’Connor P.J., Dishman R.K. (2012). Effect of exercise training on depressive symptoms among patients with a chronic illness: A systematic review and meta-analysis of randomized controlled trials. Arch. Intern. Med..

[40] Stonerock G.L., Hoffman B.M., Smith P.J., Blumenthal J.A. (2015). Exercise as Treatment for Anxiety: Systematic Review and Analysis. Ann. Behav. Med..

[41] Wegner M., Helmich I., Machado S., Nardi A.E., Arias-Carrion O. (2014). Effects of exercise on anxiety and depression disorders: Review of meta- analyses and neurobiological mechanisms. CNS Neurol. Disord. Drug Targets.

[42] Wang C.W., Chan C.H., Ho R.T., Chan J.S., Ng S.M. (2014). Managing stress and anxiety through qigong exercise in healthy adults: A systematic review and meta-analysis of randomized controlled trials. BMC Complement. Altern Med..

[43] Bartley C.A., Hay M., Bloch M.H. (2013). Meta-analysis: Aerobic exercise for the treatment of anxiety disorders. Prog Neuropsychopharmacol. Biol. Psychiatry.

[44] Heyman E., Gamelin F.X., Goekint M., Piscitelli F., Roelands B. (2012). Intense exercise increases circulating endocannabinoid and BDNF levels in humans-possible implications for reward and depression. Psychoneuroendocrinology.

[45] Archer T., Josefsson T., Lindwall M. (2014). Effects of physical exercise on depressive symptoms and biomarkers in depression. CNS Neurol. Disord Drug Targets.

[46] Greenwood B.N., Fleshner M. (2011). Exercise, Stress Resistance, and Central Serotonergic Systems. Exerc. Sport Sci. Rev..

